# A Low-Cost Gelatin-Based Model for Ultrasound-Guided Percutaneous Renal Access

**DOI:** 10.5152/tud.2025.24018

**Published:** 2025-06-04

**Authors:** Enrique Pulido-Contreras, Hedler Olaf Gonzalez-Villegas, Jorge Alejandro Lopez-Hernandez, Javier Medrano-Sanchez, Miguel Angel Garcia-Padilla

**Affiliations:** 1 Department of Urology, Unidad Médica de Alta Especialidad No. 1 Bajío, Instituto Mexicano del Seguro Social, Guanajuato, México.; 2 Department of Urology, Unidad Médica de Alta Especialidad No. 1 Bajío, Instituto Mexicano del Seguro Social and Department of Medicine and Nutrition, Universidad de Guanajuato, Guanajuato, México.; 3 Health Education Division, Unidad Médica de Alta Especialidad No. 1 Bajío, Instituto Mexicano del Seguro Social, Guanajuato, México.

**Keywords:** Gelatin-based model, percutaneous nephrolithotomy, ultrasound-guided percutaneous renal access

## Abstract

**Objective:**

The aim of this study was to develop and validate a low-cost and reproducible training model using gelatin that can be useful for acquiring the necessary skills for successful ultrasound-guided access in percutaneous nephrolithotomy without exposing patients to clinical risks.

**Methods:**

A prospective and analytical study involving 12 urology residents was conducted to validate the model using content and construct validation systems. The study consisted of 2 parts: content validation through expert opinion surveys and construct validation through resident skill assessments before and after training on the model. This model imitates the calyx to puncture and was developed using pitted olives in dense gelatin. The model was constructed for a total cost of $29.7 per unit with an easy and reproducible construction. Each model can be used to perform approximately 40 punctures before the image quality deteriorates.

**Results:**

Globally, this training model increased skills. Puncture time decreased from 106 to 40.5 seconds after training (*P* = .002). The study found that the model was accepted by 71.4% of urologists as a safe training alternative and provided a risk-free environment for practicing the 2 skills required for a successful puncture: adjusting the ultrasound machine for an adequate image of the target and surrounding tissues and needle-probe alignment.

**Conclusion:**

This model is easy to build, reproducible, and cost-effective. It enhances the residents’ skills to increase success and safety when performing ultrasound-guided percutaneous access.

## Introduction

Percutaneous nephrolithotomy (PCNL) is the treatment of choice for stones larger than 2 cm.^1^^,2^ Puncture for percutaneous renal access (PRA) is the most critical step in PCNL; however, to achieve adequate ultrasound-guided puncture, the literature recommends a minimum learning curve of 15-20 cases.^3^^-^^5^ Therefore, it is essential to implement strategies that promote technical development without exposing patients to clinical risk.

In the last decade, biological and non-biological models have been developed to facilitate this learning curve.^6^ This method is a safe and effective way to acquire new skills, with promising results.^2^ Trained students perform better than untrained students in clinical exams and invasive procedures using ultrasound phantom models.^7^

Models constructed from animal organs and plaster to simulate stones have been developed to promote skill development and proper techniques for inexperienced urologists.^2^,^8^,^9^ In addition, the use of ultrasound for PRA has been extensively shown to reduce surgical complications.^10^

This study aimed to develop and validate a low-cost, non-biologic, gelatin-based model for training in ultrasound-guided puncture for PCNL.

## Material and Methods

A prospective and analytical study including 12 urology residents was conducted to validate the model using content and construct validation systems. To achieve this goal, the study was divided into 2 parts. In the first part, the validation method described by Shepard was followed.^11^ The model was tested by 7 urologists who performed PCNL with ultrasound-guided punctures. Their experience and opinions after using the model were collected through an 8-question Likert-type survey in which the urologists described the strength and intensity of their experiences Table 1. This process involves expert opinions and is crucial in ensuring the accuracy and reliability of the model.^12^ The model was then used by residents to assess their performance before and after performing the procedure on the model for the first time, and their results were then compared with those obtained by the urologist. This procedure provided construct validation of the model and formed the second part of the study.

**Figure 1.**
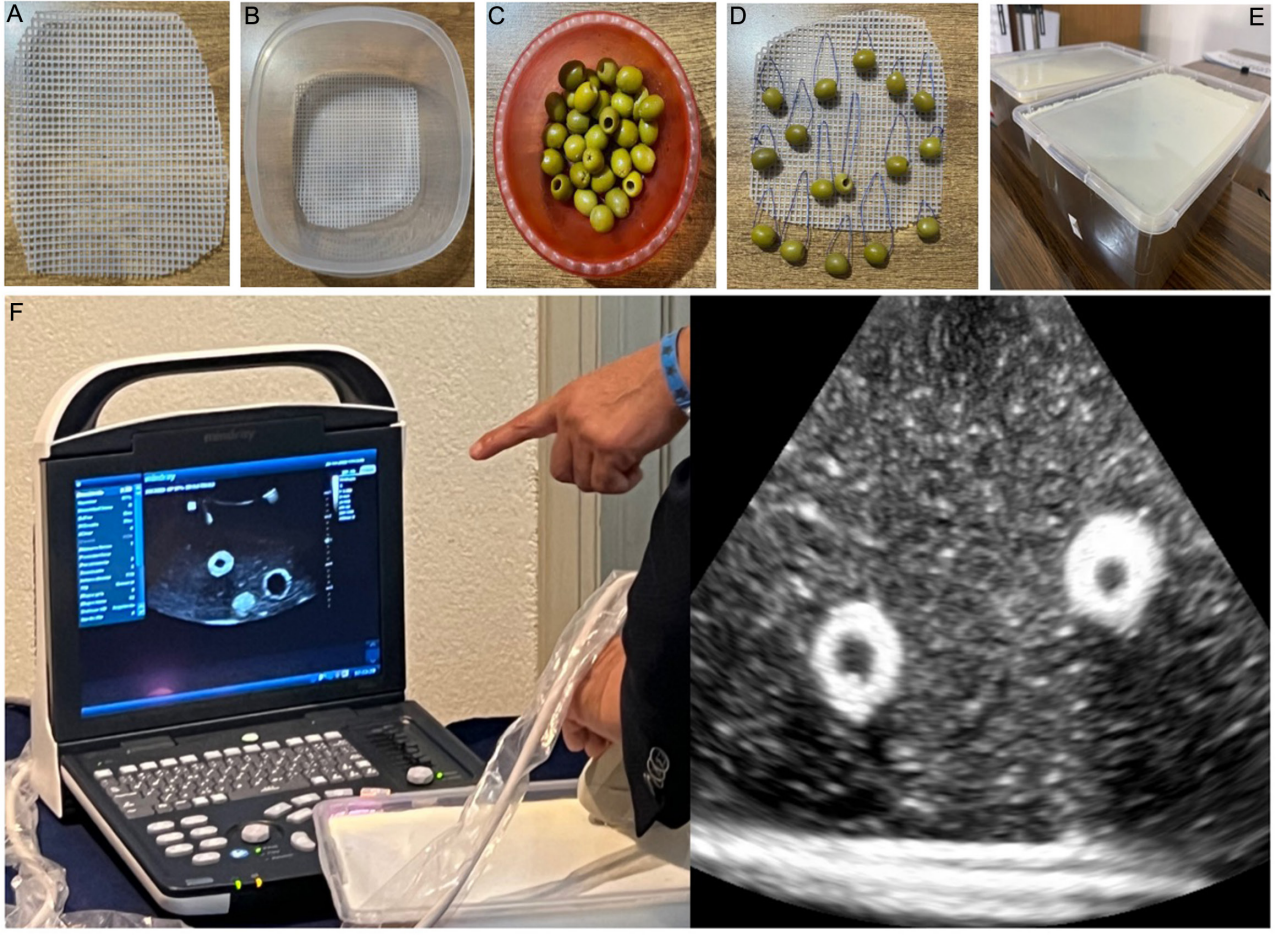
Model building. (A) Plastic mesh suited for the area of the inner base of the container. (B) Adjust the size of the plastic mesh to fit the container base. (C) Pitted olives. (D) Thread the olive’s interior with a piece of string and knot it to the plastic mesh to prevent floating when gelatin is poured; each olive represents a target. (E) For dense gelatin concentration, use 160g gelatin (300 bloom) per liter of water. Boil, stir, and add 1 tablespoon Psyllium-Plantago. Pour into a container and cover olives at the base until 8 cm thick. When the mixture is firm, apply a thin layer of liquid latex to the surface. (f) Ultrasound image of the model to puncture.

The training model used olives to create an anechoic zone representing 4 mm targets, simulating puncturing the calyx, this choice is based on their ability to generate an easily visible anechoic zone with smooth margins for puncture. The other required materials are listed in Table 2, and the model building is described in Figure 1.

At the end of the study, the aspects outlined in Table 1 had an acceptance rate of over 88%, forming the basis for the content validation of the model while ensuring participant anonymity.

Two measurements were taken for each inexperienced subject before and after using the model. The first measurement was taken when the model was used for the first time, and the second measurement was taken 24 hours after the model training was completed. The training period consisted of a 20-minute practice session with the model each day for 5 consecutive days.

Each practice session comprises the following:

1. Turn on the ultrasound machine.
2. Adjustment of parameters.
a. Frequency: 3.5MHz.
b. Focus: On the work site.
c. Depth: Adjust Zoom.
d. Dynamic range: Between 60 and 80 dB.
e. Gains and TGC: To achieve a good image.
f. Ultrasound scanning.
g. Ultrasound-guided puncture.

The urology residents received instructions from a urologist on configuring, scanning, and practicing free-hand puncture.

Finally, the initial measurements of the inexperienced group were compared with their final measurements. Similarly, these final measurements were compared with those obtained by the urologist group and the time for successful puncture between each group.

## Results

**Figure 2.**
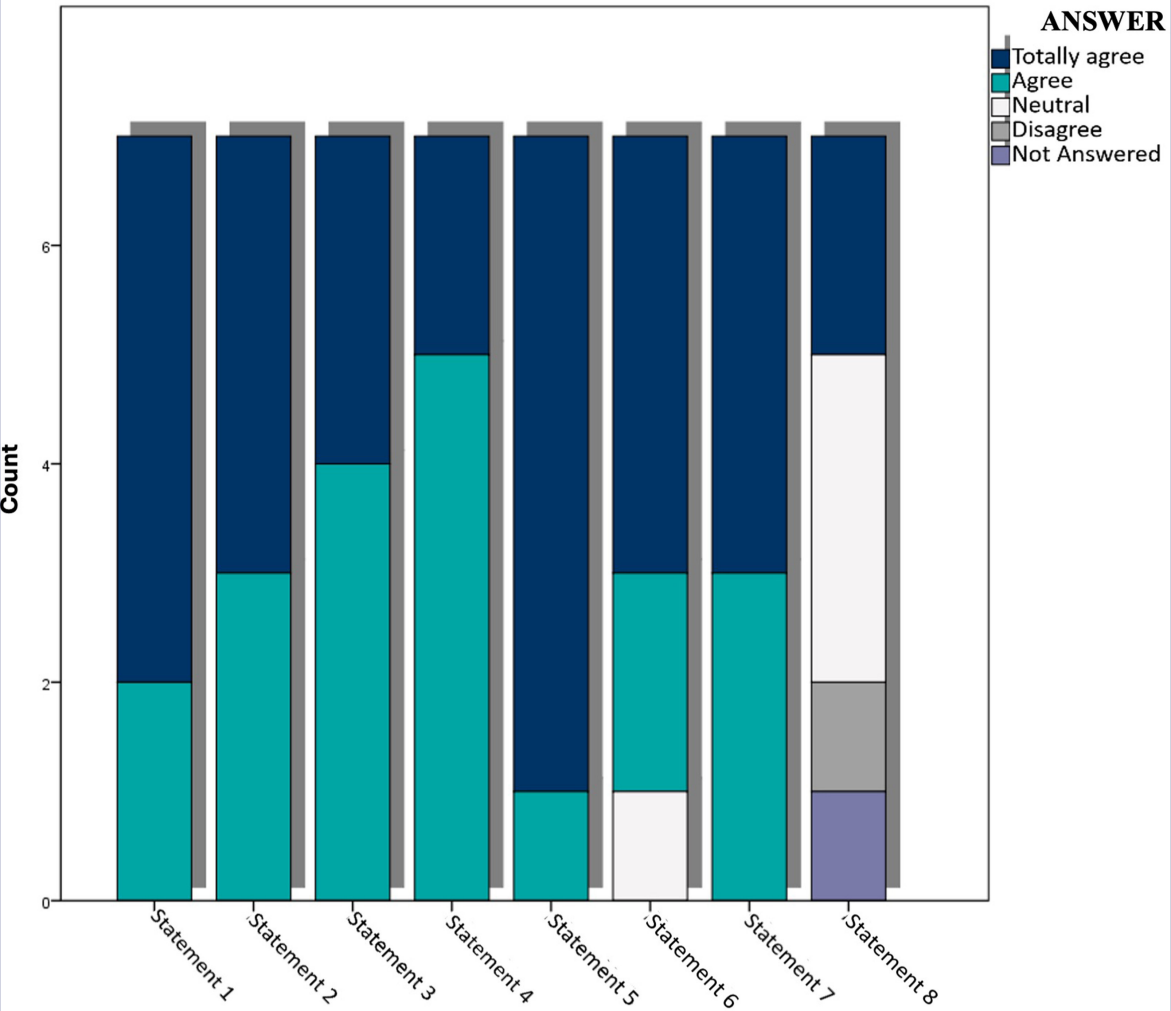
Bar chart: the frequency of responses obtained from experts for validating the training model for ultrasound-guided percutaneous renal access.

According to the observations, 71.4% of the urologists agreed that the model provides a safe training alternative, whereas 57.1% believed that it bridges the gap between classroom training and real-life medical cases, and 85.7% agreed that the model offers a platform for users to perform different approaches and techniques in a risk-free environment. Figure 2.

Simultaneously, various skills were evaluated in the model from a group of urology residents (n = 12). The 4 assessed skills were as follows: Turning on the ultrasound machine, where 8.3% and 100% of participants successfully completed the task before and after training, respectively. The second skill was parameter adjustment in the ultrasound machine to obtain an adequate image; there were no statistical differences in the ability to adjust parameters before and after training, as approximately 50% of participants partially fulfilled the task. The third skill was ultrasound scanning; before training, 75% of participants did not have prior experience with ultrasound or only partially performed ultrasound scanning; however, at the end of the training, 100% of the participants successfully completed this task. The fourth skill was performing a successful ultrasound-guided puncture; before training, no one could complete this task; after training, 50% of participants partially completed the task, and the other 50% completed the task.

The time taken to perform the puncture was measured. The mean estimated puncture time before training was 106 seconds, whereas after training, the mean decreased to 40.5 seconds (*P* = .002) Table 3.

Skills were assessed on the model between a group of urologists (n = 7) and residents (n = 12) after training. All participants (100%) successfully completed the ultrasound-guided puncture, and the puncture time was significantly shorter, with a mean of 19.1 s compared with 40.5 s for inexperienced participants. Table 4.

## Discussion

To improve expertise in ultrasound-guided PCNL, a non-biological and cost-effective model was developed and validated. This model enables stress-free training and familiarizes users with imaging and ultrasound equipment. Simulated practice is increasingly used in health education to acquire and maintain skills.^13^ The development of expertise requires deliberate and sustained practice, making this learning strategy crucial.^14^ However, it is currently considered ethically unacceptable for inexperienced learners to practice with real patients to develop their skills. In addition, technological advances have significantly transformed procedures in the surgical field, as the advent of minimally invasive surgery and ultrasound-guided procedures demands adaptation by professionals and trainee residents to these technologies.^13^,^14^ The lack of experience in certain areas often leads to high-risk situations and complications.^13^,^15^ Ali developed a 3D-printed model using polylactic acid filament based on the renal and pyelocaliceal anatomy from contrast-enhanced tomography of a patient with nephrolithiasis.^2^ They used this model with a group of novices and compared their results with those obtained by a second group using the URO Mentor^TM^ surgical simulator. The group using the non-biological model demonstrated better outcomes and superior results for PRA. Bozzini, employing a similar construction model that included the anatomy of the posterior abdominal wall, demonstrated similar results with a larger sample of approximately 110 novices.^16^ Karagozlu conducted a study that was similar in structure to ours. Their study presented a model comprising 3D-printed collecting system prototypes inside a rubber and foam box to urologists and novice groups. After continuous use, they confirmed that the participants had acquired the necessary skills to perform PRA with fluoroscopic guidance.^9^ However, a literature search found no evidence regarding using non-biological gelatin-based models for skill acquisition in PRA. This model achieved similar results (*P* < .001) to those obtained with 3D-printed models because the quality of the image observed with ultrasound guidance is crucial for a successful puncture.^2^,^9^

Our study found that the residents could acquire new skills effectively using the model. The residents demonstrated their expertise in ultrasound scanning by accurately performing the puncture and reducing the time required for a successful puncture. These 3 parameters are critical in a professional setting, such as the operating room. This accomplishment is like the skill acquisition demonstrated by the evaluated groups in the studies mentioned.^2^,^8^,^9^,^16^

Now that this model is comparable to existing models with the same purpose,^2^^,^^8^^,^^9^ one of its strengths is its affordable cost due to the simplicity of the materials needed for its construction. This aspect is significant because it can provide coverage to a large trainee population in hospitals that cannot afford expensive models. Several studies with tasks and skills like those used to validate this model have been conducted recently. These studies used more advanced non-biological models and obtained the same significant *P*-values for tasks such as ultrasound scanning of the calyx to be punctured and the time required to perform the puncture.^2^^,^^9^ Similarly, in studies with a study design like ours, the sample of urologists was subjected to an evaluation questionnaire after using the model that provided approving ratings for statements, as presented in Table 1. However, these studies achieved much better scores for the anatomical variation of the model and the approach to different pathologies.^2^^,^^11^^,^^9^ Because ultrasound-guided puncture remains one of the primary objectives of this model, it has emerged as a safe, valid, and affordable alternative for practice in this approach. The urologists should perform their percutaneous access. A study by Watterson found lower complications (5 vs. 15; *P* = .02) and a higher stone-free rate (86% vs. 61%; *P* = .01) when PRA was performed by a urologist instead of a radiologist.^17^

Finally, the current model is subject to certain limitations. Although the tissue sensation was satisfactory in the study’s first phase, it falls short compared to real-life scenarios. The container and gelatin used in the construction did not resemble human renal anatomy or a collecting system. Therefore, this model cannot simulate renal mobilization and complications. The number of study participants was limited. The aim was to increase the sample size in future studies.

This model for practicing percutaneous renal puncture is easy to build, reproducible, and cost-effective, significantly enhancing the skills of the resident to increase success and safety when performing ultrasound-guided percutaneous access in patients.

## Figures and Tables

**Table 1.** Statements from the Expert Survey for the Validation of the Training Model for Ultrasound-Guided Percutaneous Nephrolithotomy

Statement 1	The model provides a safe training alternative for real-life cases.
Statement 2	The model serves as a bridge between theoretical training conducted in classrooms and practical medical cases encountered in real-life situations.
Statement 3	Model simulation allows the evaluation of student performance.
Statement 4	The model provides a platform for surgeon certification.
Statement 5	The model offers a platform for users to perform different approaches and techniques in a risk-free environment.
Statement 6	The texture of the interaction is sufficiently realistic.
Statement 7	The model provides clinically relevant objective measurements that can be used to evaluate user performance.
Statement 8	The model allows variation in different anatomies or pathologies through interchangeable parts.

**Table 2.** Materials Needed for Model Construction

Material	Price
Pitted olives	$1.3, 70 gr bag
Extra-firm gelatin (>300 blooms)	$14, 1 kg bag
Psylium plantago	$7, 500 gr bag
Silicone or liquid latex	$0.6, 30 ml bottle
Plastic mesh	$0.9 per piece
Thread	$1.1 per piece
Container	$4,8 with a 3 lt capacity.
Total	$29.7 at the local currency exchange rate in December 2023

**Table 3.** Comparison of Skills Before and After the Use of the Model by a Group of Novices; Initial Measurements vs. Final Measurements, n = 12

Skill	Before	After	*P*
Placing or turning on the model^a^	4 (33.3) 7 (58.3) 1 (8.3)		<.001
Not completed		0	
Partially completed		0	
Completed		12 (100)	
Parameters adjustment^b^	4 (33.3) 6 (50) 2 (16.7)		.054
Not completed		1 (8.3)	
Partially completed		5 (41.7)	
Completed		6 (50)	
Ultrasound scan ^c^	3 (25) 6 (50) 3 (25)		.001
Not completed		0	
Partially completed		0	
Completed		12 (100)	
Ultrasound-guided puncture^d^	6 (50) 6 (50) 0		.001
Not completed		0	
Partially completed		6 (50)	
Completed		6 (50)	
Puncture time (seconds) ^e^	106.5 ± 69.2	40.5 (22.2-121.7)	.002

Values presented in frequency and percentage, *P*-value obtained by the chi-square test for linear trend.

^a^It is considered partially completed when the participant was able to turn on the machine but cannot use the probe, completed when they can use the ultrasound probe.

^b^It is considered completed when the participant was able to adjust all the parameters and partially completed when only adjusted 2 to 4.

^c^It is considered completed when the participant was able to scan all the targets and partially completed when only scanned some targets.

^d^It is considered a completed or successful puncture when the needle was inside the target and allowed for the mobilization of the olive, partially completed when the puncture was in the olive but not in the center.

^e^Values presented as mean ± SD; median (percentile 25-75); *P*-value obtained by Mann–Whitney *U* test.

**Table 4.** Validation of the Model Through the Differences in Skills Between the Inexperienced Group and Expert Doctors

Skills	Inexperienced Group After Use of the Model, n = 12	Group of Urologists n = 7	*P*
Placing or turning on the model	0 0 12 (100)		^–^
Not completed		0	
Partially completed		0	
Completed		7 (100)	
Parameters adjustment	1 (8.3) 5 (41.7) 6 (50)		.306 ^b^
Not completed		0	
Partially completed		2 (28.6)	
Completed		5 (71.4)	
Ultrasound scan	0 0 12 (100)		–
Not completed		0	
Partially completed		0	
Completed		7 (100)	
Ultrasound-guided puncture ^d^	0 6 (50) 6 (50)	0 0 7 (100)	.044 ^a^
Not completed			
Partially completed			
Completed			
Puncture time (seconds) ^c^	40.5 (22.2 – 121.7)	19.1 ± 6.9	.018

Values presented in frequency and percentage.

^a^*P*-value obtained using Fisher’s exact test.

^b^*P*-value obtained by the chi-square test for linear trend.

^c^Values presented with median (percentile 25-75); mean ± SD; *P*-value obtained by Mann–Whitney *U* test.

^d^It is considered a completed or successful puncture when the needle was inside the target and allowed for the mobilization of the olive.

## Data Availability

The data that support the findings of this study are available on request from the corresponding author.

## References

[1] Skolarikos A Jung H Neisius A EAU Guidelines on Urolithiasis. Edn. presented at the EAU Annual Congress Milan 2023. ISBN 978-94-92671-19-6. EAU Guidelines Office, Arnhem, The Netherlands. 2023. Available at: http://uroweb.org/guidelines/compilations-of-all-guidelines/.

[2] Ali S Sirota E Ali H Three-dimensionally printed non-biological simulator for percutaneous nephrolithotomy training. Scand J Urol. 2020;54(4):349 354. (doi: 10.10.1080/21681805.2020.1773529) 32496922

[3] Pulido-Contreras E Garcia-Padilla MA Medrano-Sanchez J Leon-Verdin G Primo-Rivera MA Sur RL. Percutaneous nephrolithotomy with ultrasound-assisted puncture: does the technique reduce dependence on fluoroscopic ionizing radiation? World J Urol. 2021;39(9):3579 3585. (doi: 10.10.1007/s00345-021-03636-2) 33646346

[4] Song Y Ma Y Song Y Fei X. Evaluating the learning curve for percutaneous nephrolithotomy under total ultrasound guidance. PLoS One. 2015;10(8):e0132986. (doi: 10.10.1371/journal.pone.0132986) PMC4535977 26271037

[5] Usawachintachit M Masic S Allen IE Li J Chi T. Adopting ultrasound guidance for prone percutaneous nephrolithotomy: evaluating the learning curve for the experienced surgeon. J Endourol. 2016;30(8):856 863. (doi: 10.10.1089/end.2016.0241) 27150671 PMC4964751

[6] Stern J Zeltser IS Pearle MS. Percutaneous renal access simulators. J Endourol. 2007;21(3):270 273. (doi: 10.10.1089/end.2007.9981) 17444770

[7] Schwartz CM Ivancic RJ McDermott SM Bahner DP. Designing a low-cost thyroid ultrasound phantom for medical student education. Ultrasound Med Biol. 2020;46(6):1545 1550. (doi: 10.10.1016/j.ultrasmedbio.2020.01.033) 32143859

[8] Vijayakumar M Balaji S Singh A Ganpule A Sabnis R Desai M. A novel biological model for training in percutaneous renal access. Arab J Urol. 2019;17(4):292 297. (doi: 10.10.1080/2090598X.2019.1642600) 31723446 PMC6830254

[9] Karagozlu AA Unal D Demirbas M A simple, non-biological model for percutaneous renal access training. Eur Urol Suppl. 2016;15(10):e1272 e1275. (doi: 10.10.1016/S1569-9056(16)30158-0) 29250765

[10] Wang K Zhang P Xu X Fan M. Ultrasonographic versus Fluoroscopic Access for Percutaneous Nephrolithotomy: a Meta-Analysis. Urol Int. 2015;95(1):15 25. (doi: 10.10.1159/000369216) 25678305

[11] Shepard L Schuler N Saxton A Use of 3D printing and hydrogel molding to develop a model for ultrasound-guided percutaneous nephrolithotomy (PCNL) training and education. Urology Video Journal. 2023;18:100216. (doi: 10.10.1016/j.urolvj.2023.100216)

[12] Gallagher AG Ritter EM Satava RM. Fundamental principles of validation, and reliability: rigorous science for the assessment of surgical education and training. Surg Endosc. 2003;17(10):1525 1529. (doi: 10.10.1007/s00464-003-0035-4) 14502403

[13] Motola I Devine LA Chung HS Sullivan JE Issenberg SB. Simulation in healthcare education: a best evidence practical guide. AMEE Guide No. 82. Med Teach. 2013;35(10):e1511 e1530. (doi: 10.10.3109/0142159X.2013.818632) 23941678

[14] Evgeniou E Loizou P. Simulation-based surgical education. ANZ J Surg. 2013;83(9):619 623. (doi: 10.10.1111/j.1445-2197.2012.06315.x) 23088646

[15] Erdemir A Mulugeta L Ku JP Credible practice of modeling and simulation in healthcare: ten rules from a multidisciplinary perspective. J Transl Med. 2020;18(1):369. (doi: 10.10.1186/s12967-020-02540-4) PMC7526418 32993675

[16] Bozzini G Maltagliati M Berti L Development and Validation of a Novel Skills Training Model for PCNL, an ESUT project. Acta Biomed. 2022;93(4):e2022254. (doi: 10.10.23750/abm.v93i4.11821) 36043983 PMC9534240

[17] Watterson JD Soon S Jana K. Access related complications during percutaneous nephrolithotomy: urology versus radiology at a single academic institution. J Urol. 2006;176(1):142 145. (doi: 10.10.1016/S0022-5347(06)00489-7) 16753389

